# Agreement in reporting of asthma by parents or offspring – the RHINESSA generation study

**DOI:** 10.1186/s12890-018-0687-4

**Published:** 2018-07-27

**Authors:** Ingrid N. Kuiper, Cecilie Svanes, Bryndis Benediktsdottir, Randi J. Bertelsen, Lennart Bråbäck, Shyamali C. Dharmage, Mathias Holm, Christer Janson, Rain Jögi, Andrei Malinovschi, Melanie Matheson, Jesús Martínez Moratalla, Francisco Gómez Real, José Luis Sánchez-Ramos, Vivi Schlünssen, Signe Timm, Ane Johannessen

**Affiliations:** 1 Department of Occupational Medicine, Haukeland University Hospital, N-5021 Bergen, Norway; 2 Centre for International Health, Department of Global Public Health and Primary Care, University of Bergen, Bergen, Norway; 3 Faculty of medicine, University of Iceland, Reykjavik, Iceland; 4 Occupational and Environmental Medicine, Umeå University, Umeå, Sweden; 5 Allergy and Lung Health Unit, Melbourne School of Population and Global Health, University of Melbourne, Melbourne, Australia; 6 Department of Occupational and Environmental Medicine, University of Gothenburg, Gothenburg, Sweden; 7 Department of Medical Sciences: Respiratory Allergy and Sleep Research, Uppsala University, Uppsala, Sweden; 8 Lung Clinic, Tartu University Hospital, Tartu, Estonia; 9 Department of Medical Sciences: Clinical Physiology, Uppsala University, Uppsala, Sweden; 10 Servicio de Salud de Castilla, Servicio de Neumología del Complejo Hospitalario Univerisitario de Albacete, La Mancha, Albacete, Spain; 11 Department of Clinical Science, University of Bergen, Bergen, Norway; 12 Department of Gynecology and Obstetrics, Haukeland University Hospital, Bergen, Norway; 13 Facultad de Enfermeria, University of Huelva, Huelva, Spain; 14 Department of Public Health, Danish Ramazzini Center, Aarhus University, Aarhus, Denmark; 15 National Research Center for the Working Environment, Copenhagen, Denmark

**Keywords:** Agreement, Validation, Asthma, Questionnaire, Self-report, Transgenerational

## Abstract

**Background:**

Self-report questionnaires are commonly used in epidemiology, but may be susceptible to misclassification, especially if answers are given on behalf of others, e.g. children or parents. The aim was to determine agreement and analyse predictors of disagreement in parents’ reports of offspring asthma, and in offspring reports of parents’ asthma.

**Methods:**

In the Respiratory Health in Northern Europe, Spain and Australia (RHINESSA) generation study, 6752 offspring (age range 18–51 years) and their parents (age range 39–66 years) reported their own and each other’s asthma status. Agreement between asthma reports from offspring and parents was determined by calculating sensitivity, specificity, positive and negative predictive value and Cohen’s kappa. The participants’ own answers regarding themselves were defined as the gold standard. To investigate predictors for disagreement logistic regression analyses were performed to obtain odds ratios (OR) with 95% confidence intervals (CI) for sex, smoking status, education, comorbidity and severity of asthma.

**Results:**

Agreement was good for parental report of offspring early onset asthma (< 10 years, Cohen’s kappa 0.72) and moderate for offspring later onset asthma (Cohen’s kappa 0.46). Specificity was 0.99 for both, and sensitivity was 0.68 and 0.36, respectively. For offspring report of maternal and paternal asthma the agreement was good (Cohen’s kappa 0.69 and 0.68), specificity was 0.96 and 0.97, and sensitivity was 0.72 and 0.68, respectively. The positive predictive value (PPV) was lowest for offspring report of maternal asthma (0.75), and highest for parents’ report of early onset asthma in the offspring (0.83). The negative predictive value (NPV) was high for all four groups (0.94–0.97). In multivariate analyses current smokers (OR = 1.46 [95% CI 1.05, 2.02]) and fathers (OR = 1.31 [95% CI 1.08, 1.59]) were more likely to report offspring asthma incorrectly. Offspring wheeze was associated with reporting parental asthma incorrectly (OR = 1.60 [95% CI 1.21, 2.11]), both under- and over reporting.

**Conclusions:**

Asthma reports across generations show moderate to good agreement, making information from other generations a useful tool in the absence of direct reports.

**Electronic supplementary material:**

The online version of this article (10.1186/s12890-018-0687-4) contains supplementary material, which is available to authorized users.

## Background

Asthma is the most common non-communicable disease among children and one of the most prevalent chronic diseases worldwide [1, 2]. The World Health Organization [3] estimated in 2011 that 235 million people suffer from asthma, but later studies have indicated numbers as high as 300 million and projected that the worldwide prevalence will increase to 400 million by 2025 [4].

The investigation of asthma is complex. Questionnaires are often preferred in epidemiological studies to determine disease occurrence because they are cost-efficient and simple to perform compared to clinical examination. Questionnaire data on the prevalence of asthma have been used for epidemiological research since the mid 1960s. In 1995 the International Study of Asthma and Allergies in Childhood [5] developed a standardized questionnaire to improve the investigation of asthma in an epidemiological setting [5–7]. Even though clinical examination has been regarded as the gold standard for assessing asthma, recent studies show that questionnaires are a useful epidemiological tool, being reasonably valid [8–10].

Nevertheless, self-reported information is susceptible to misclassification such as recall bias, and it may be particularly susceptible to misclassification if participants are asked to provide information on behalf of others, for example their children or their parents [11, 12]. At the same time, in absence of direct reports, asthma reports on behalf of family members can be highly valuable in a clinical setting if the patient cannot report on behalf of himself/herself or in absence of prior patient history. However, validity of such intergenerational reports with regard to asthma has been poorly investigated, mostly focusing on current asthma status asked at the same time, and only including young children and adolescents [13, 14]. With an increasing interest in intergenerational risk factors [15–19] more attention should be given to validate this kind of information across generations.

The Respiratory Health in Northern Europe, Spain and Australia generation study (RHINESSA) uses questionnaires, interviews, and clinical examinations to study asthma and lung health throughout the lifespan and across generations. Participants are asked to provide information about themselves as well as their children and parents. To underpin the research of RHINESSA and to shed light on this important part of epidemiological methodology, the aim of the present paper was to assess agreement between parental report of offspring asthma as compared to offspring’s own report, to assess agreement between offspring’s report of parent’s asthma as compared to parents’ own report, and to investigate predictors for discrepant answers.

## Methods

### Study design and population

This agreement study compares questionnaires about asthma from two generations. The primary sources of data are parents from the Respiratory Health in Northern Europe study (RHINE, www.rhine.nu) and the European Community Respiratory Health Survey (ECRHS, www.ecrhs.org) and their offspring included in the RHINESSA study (www.rhinessa.net). The parent and offspring pairs provided information on asthma status regarding both themselves and each other.

#### Parent population

RHINE is a prospective questionnaire-based cohort study comprising subjects from seven Northern European centres: Reykjavik (Iceland), Bergen (Norway), Umea, Uppsala and Gothenburg (Sweden), Aarhus (Denmark) and Tartu (Estonia). All subjects participated in stage 1 of the ECRHS I in 1990, together with many other centres, among others Melbourne (Australia) and Huelva and Albacete (Spain) [20, 21]. Both ECRHS and RHINE had follow-ups after 10 and 20 years, and have investigated incidence, prevalence and risk factors for respiratory diseases, allergies and symptoms related to asthma and chronic obstructive pulmonary disease (COPD) throughout this time period. Response rate in RHINE III was 61%. The ECRHS subjects from the Spanish and Australian study centres filled in the ECRHS stage 3 screening questionnaire; which includes identical questions as in RHINE III for all characteristics needed in the present study.

#### Offspring population

In the period 2013–2015 questionnaires were sent to all adult offspring (> 18 years) of parents from the RHINE centres and the Spanish and Australian ECRHS centres, and a sub-sample was invited for clinical examination. The questionnaires were web-based in all centres except Sweden where they used postal questionnaires. Overall response rate was 33.5%, varying across centres from 18.6% in Tartu to 73.7% in Melbourne (See Additional file 1: Table S1).

### Predictors and outcomes

The main outcome in this agreement study was physician diagnosed asthma self-reported by the participants. Reports of one’s own asthma were “doctors-diagnosed asthma” while reports of asthma in others were “ever asthma”. In more detail, the asthma outcomes were built on the following wordings:

Parents reported doctor-diagnosed asthma about themselves by answering yes to the questions “Do you have or have you ever had asthma?” and “Has it been confirmed by a medical doctor?”

Parents reported asthma about their offspring by answering yes to the questions “For each child, please tick yes if they had asthma before 10 years” and/or “For each child, please tick yes if they had asthma after 10 years”. The former was classified as early onset asthma in the offspring, while the latter was classified as late onset asthma.

Offspring reported doctor-diagnosed asthma about themselves by answering yes to the questions “Do you have or have you ever had asthma?” and “Has it been confirmed by a medical doctor?”

Offspring were also asked how old they were when they first experienced asthma symptoms.

Offspring reported asthma about their parents defined by answering yes to the questions “Did your mother ever suffer from asthma?” and “Did your father ever suffer from asthma?”. Questionnaires are available from www.rhinessa.net.

A discrepant asthma report by parents was defined as parents reporting absence of asthma in their offspring when the offspring report presence of asthma, or parents reporting presence of asthma in their offspring when the offspring state they do not have asthma. In the same manner, a discrepant asthma report by offspring was defined as offspring reporting absence of asthma in their parents when the parents report presence of asthma, or offspring reporting presence of asthma in their parents when the parents themselves state they do not have asthma.

Predictors for discrepant reports between parent and offspring pairs were investigated for the following covariates in each generation: smoking status (never-, ex- or current smoker), education (primary school, secondary school or college/university), respiratory symptoms (wheeze, wheeze with shortness of breath, awoken with tightness in chest, awoken with attack of cough in the past 12 months and currently taking medication) and comorbidities (hypertension, stroke, ischemic heart disease, diabetes mellitus, COPD and serious respiratory infections before the age of 5 years).

### Statistical analyses

All analyses were performed using Stata version 14.0.

The overall agreement between asthma reports from offspring and parents was calculated by Cohen’s kappa. The following interpretation categories were used: poor agreement, < 0.2; fair, 0.21–0.40; moderate, 0.41–0.60; good, 0.61–0.80; and very good, 0.81–1.00 [22]. Sensitivity, specificity and predictive values were calculated using the participants’ own answers regarding themselves as the golden standard. Descriptive analyses and estimations of Cohen’s kappa, sensitivity, specificity and predictive values were performed stratified by sex to investigate any specific differences between mothers and fathers, and between daughters and sons.

We performed univariate logistic regressions with each covariate as predictor and discrepant report (yes/no) in parent-offspring pairs as outcome. The participants’ own answers regarding themselves were considered the gold standard also in these analyses. Significant predictors (*p* < 0.05) from the univariate analyses were carried forward to multivariate logistic regression. Separate models were constructed for discrepant reports by parents and discrepant reports by offspring. In addition, the multivariate analyses were adjusted for study centre and sibling status (siblings in RHINESSA/no siblings in RHINESSA).

### Ethical approval

In all study centres written informed consent was obtained from each participant, and the study was approved by regional committees of medical research ethics in each study centre according to national legislations.

## Results

Reports of 6752 offspring and their parents who had answered questions regarding their own and each other’s asthma status were included from the ten study centres. Table 1 shows the characteristics of the study population by parents and offspring. A slight majority of the population was female. The mean age for the mothers and fathers were 54.0 (±6.5) and 54.7 (±6.1) respectively. More mothers than fathers and more daughters than sons reported having asthma. For the offspring, mean age was 30 years and did not differ significantly between males and females. More mothers than fathers and more daughters than sons had obtained university education. Slightly more mothers than fathers were current smokers, whereas in offspring more sons than daughters smoked. The reports of respiratory symptoms were comparable across gender for the parents, but in the offspring, all respiratory symptoms, except the symptoms awoken with attack of breathlessness and wheeze with shortness of breath, were significantly higher for the daughters (Table 1).

**Table 1:** Study population characteristics, 5907 parents and 6752 offspring included in the RHINESSA generation study

Characteristics	Parents (RHINE/ECRHS)			Children (RHINESSA)		
	Mother	Father	P^a^	Daughter	Son	P^a^
	N (%)	N (%)		N (%)	N (%)	
N (%)	3377 (57)	2530 (43)		3910 (58)	2842 (42)	
Albacete	39 (57)	30 (43)		39 (53)	34 (47)	
Bergen	593 (53)	518 (47)		706 (60)	480 (40)	
Gothenburg	413 (56)	322 (44)		491 (53)	439 (47)	
Huelva	42 (69)	19 (31)		44 (64)	25 (36)	
Melbourne	96 (55)	80 (45)		42 (62)	26 (38)	
Reykjavik	409 (55)	328 (45)		543 (61)	342 (39)	
Tartu	205 (68)	97 (32)		177 (60)	119 (40)	
Umea	629 (60)	423 (40)		735 (57)	545 (43)	
Uppsala	602 (59)	420 (41)		713 (56)	556 (44)	
Aarhus	349 (54)	293 (46)		420 (60)	276 (40)	
Mean age (SD)	54.0 (6.5)	54.7 (6.1)	**< 0.001**	30.3 (7.7)	30.4 (7.8)	0.930
Asthma (%)	478 (14)	292 (12)	**0.004**	671 (17)	420 (15)	**0.008**
Smoking			**0.009**			**< 0.001**
Never-smokers	1496 (46)	1155 (47)		2602 (67)	1942 (69)	
Ex-smokers	1400 (43)	1100 (45)		850 (22)	503 (18)	
Current smokers	337 (10)	197 (8)		445 (11)	382 (14)	
Education			**< 0.001**			**< 0.001**
Primary school	385 (12)	296 (12)		97 (3)	91 (3)	
Secondary school	1224 (38)	1029 (44)		1282 (33)	1259 (44)	
College/university	1576 (50)	1068 (45)		2521 (65)	1487 (52)	
Parental asthma			0.058			**< 0.001**
Mother	295 (10)	206 (9)		476 (12)	272 (10)	
Father	218 (7)	124 (6)		309 (8)	179 (6)	
No one	2518 (83)	1922 (85)		3038 (79)	2333 (83)	
Both	12 (0.4)	12 (0.5)		39 (2)	23 (1)	
Other diseases^b^						
Comorbidity	892 (26)	778 (31)	**< 0.001**	165 (4)	124 (4)	0.770
Hypertension	778 (23)	659 (26)	**0.005**	120 (3)	96 (4)	0.390
Stroke	54 (2)	46 (2)	0.510	–	–	–
Ischemic heart disease	64 (2)	116 (5)	**< 0.001**	5 (0.1)	8 (0.3)	0.150
Diabetes mellitus	106 (3)	107 (5)	**0.027**	53 (1)	30 (1)	0.270
COPD	73 (2)	60 (2)	0.600	10 (0.3)	5 (0.2)	0.490
Serious childhood infection < 5 years	254 (8)	126 (5)	**< 0.001**	331 (9)	185 (7)	**< 0.001**
Respiratory symptoms			0.246			**0.001**
Wheeze^c^	669 (20)	530 (21)	0.330	697 (18)	468 (17)	0.150
Wheeze with shortness of breath^c^	421 (13)	297 (12)	0.380	457 (62)	256 (49)	**< 0.001**
Awoken with tightness in chest^c^	364 (11)	245 (10)	0.170	497 (13)	286 (10)	**< 0.001**
Awoken with attack of breathlessness^c^	220 (7)	126 (5)	**0.013**	173 (4)	136 (5)	0.48
Awoken with attack of cough^c^	1093 (33)	533 (21)	**< 0.001**	1343 (34	562 (20)	**< 0.001**
Currently taking asthma medication	327 (10)	216 (9)	0.130	383 (10)	212 (8)	**< 0.001**

^a^All *p*-values < 0.05 = significant and are marked bold. *P*-values are estimated from two-group mean comparison test (unpaired t-test) for continuous values and chi-squared test for categorical values

^b^Comorbidity includes the variables: hypertension, stroke, ischemic heart disease, diabetes mellitus, COPD, serious childhood infection < 5 years. Questions about stroke and COPD were not included in the offspring-questionnaires

^c^In the past 12 months

**Fig. 1:**
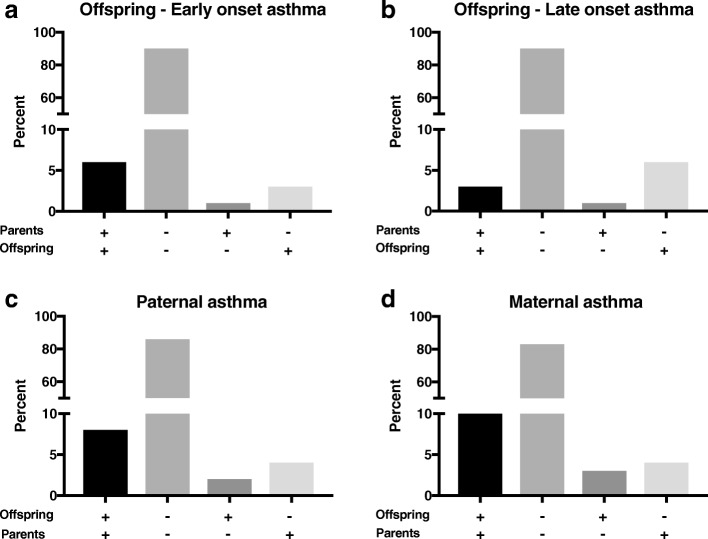
Parent-reported offspring asthma as compared to offspring’s own report (**a**, **b**); and offspring-reported paternal (**c**) and maternal (**d**) asthma as compared to parents’ own report

Figure 1 summarizes offspring early/late onset asthma and the corresponding parent report (Fig. 1a and b), as well as paternal and maternal asthma and the corresponding offspring report (Fig. 1c and d). The vast majority of parents answered correctly concerning their offspring’s asthma status: in 4798 (90%) of the reports on early onset asthma, both parents and offspring answered “no” and 323 (6%) “yes” to the presence of asthma in the offspring. The reports about late onset asthma followed the same pattern: in 4816 (90%) reports both parents and offspring answered “no asthma” in the offspring, and both parts reported late onset asthma in the offspring among 185 (3%). Parents’ discrepant answers were most often that they reported no asthma for asthmatic offspring rather than reporting asthma for non-asthmatic offspring (Fig. 1a and b).

In 86% of the cases, both offspring and fathers reported absence of paternal asthma, compared to 83% for maternal asthma status. In 8% of the reports, offspring and fathers both reported a present asthma diagnosis; the corresponding frequency for maternal asthma was 10%. It was also more common for offspring to report no asthma in asthmatic parents than to report asthma in non-asthmatic parents.

The specificity was high for all four groups (Table 2), while sensitivity was lower. The sensitivity was lowest for the late onset asthma in the offspring (0.36), and highest for the maternal asthma (0.72). The positive predictive value (PPV) was lowest for the maternal asthma (0.75), and highest for the early onset asthma in the offspring (0.83). The negative predictive value (NPV) was high for all four groups (0.94–0.97).

**Table 2:** Parameter estimates (95% CI) for Cohen’s kappa, sensitivity, specificity, PPV and NPV for offspring-reported parental asthma and parent-reported offspring asthma

Offspring asthma	Agreement^a^ N (%)	Disagreement^b^ N (%)	Cohens kappa	Sensitivity	95% CI	Specificity	95% CI	PPV	95% CI	NPV	95% CI
Early onset asthma	5121 (96)	219 (4)	0.72	0.68	0.64, 0.72	0.99	0.98, 0.99	0.83	0.79, 0.86	0.97	0.96, 0.97
Mother	2935 (96)	110 (4)	0.75	0.72	0.66, 0.77	0.99	0.98, 0.99	0.83	0.77, 0.88	0.98	0.97, 0.98
Father	2186 (95)	109 (5)	0.69	0.64	0.57, 0.70	0.99	0.98, 0.99	0.83	0.76, 0.88	0.96	0.95, 0.97
Late onset asthma	5001 (93)	376 (7)	0.46	0.36	0.32, 0.41	0.99	0.98, 0.99	0.79	0.73, 0.84	0.94	0.93, 0.94
Mother	2891 (94)	199 (6)	0.53	0.43	0.37, 0.49	0.99	0.98, 0.99	0.82	0.75, 0.88	0.94	0.93, 0.95
Father	2110 (92)	177 (8)	0.36	0.26	0.21, 0.33	0.99	0.98, 0.99	0.73	0.61, 0.82	0.93	0.92, 0.94
Parental asthma											
Maternal	3477 (93)	276 (7)	0.69	0.72	0.68, 0.75	0.96	0.95, 0.97	0.75	0.71, 0.79	0.95	0.95, 0–96
Daughter	2009 (92)	163 (8)	0.69	0.75	0.69, 0.79	0.95	0.94, 0.96	0.71	0.66, 0.76	0.96	0.95, 0.97
Son	1468 (93)	113 (7)	0.70	0.68	0.61, 0.74	0.97	0.96, 0.98	0.81	0.75, 0.86	0.95	0.93, 0.96
Paternal	2817 (94)	182 (6)	0.68	0.68	0.63, 0.73	0.97	0.96, 0.98	0.76	0.71, 0.80	0.96	0.95, 0.97
Daughter	1637 (94)	101 (6)	0.71	0.72	0.65, 0.78	0.97	0.96, 0.98	0.77	0.70, 0.83	0.96	0.95, 0.97
Son	1180 (94)	81 (6)	0.64	0.63	0.54, 0.71	0.97	0.96, 0.98	0.74	0.65, 0.82	0.96	0.94, 0.97

^a^Agreement: when both parents and offspring answered the same (yes/yes or no/no)

^b^Disagreement: when parents and offspring answered differently (yes/no or no/yes)

Agreement between the parental reports and early onset asthma in offspring was good (Cohen’s kappa 0.72), and moderate for late onset asthma in offspring (Cohen’s kappa 0.46). The agreement between offspring reports and maternal and paternal asthma were both good (Cohen’s kappa 0.69 and 0.68, respectively). Additional analyses stratified by study centres are given in the additional file (See Additional file 1: Table S2) and show some variability between the centres. To investigate if offspring reports of parental asthma differed in agreement with the parental reports according to age of asthma onset in parents, we performed an additional analysis (See Additional file 1: Table S3) stratifying by the timing of parental asthma onset. This analysis showed that offspring reported asthma in their parents more correctly if the parents had their asthma before the children were 20 years old, than if the parents got their asthma diagnosis after their offspring were 20 years old.

Table 3 shows univariate and multivariate logistic regression analyses of the association between covariates and offspring/parent discrepant asthma reports. In the univariate analyses regarding parent-reported offspring asthma, several parental factors were associated with reporting incorrect: male gender, current smoker, ex-smoker, ischemic heart disease, diabetes, COPD, wheeze and wheeze with shortness of breath. In the univariate analyses regarding the offspring-reported parental asthma, the following factors on the offspring level were associated with reporting incorrectly: ex-smoker, wheeze, currently taking asthma medication and late onset of own asthma.

**Table 3:** Odds ratios for discrepant answers in offspring/parent asthma reports, univariate and multivariate logistic regression analysis

Predictor	Univariate analyses Parents		Multivariate analyses^a^ Parents		Univariate analyses Offspring		Multivariate analyses^a^ Offspring	
	OR (95% CI)	p	OR (95% CI)	p	OR (95% CI)	p	OR (95% CI)	p
Male gender	1.33 (1.11, 1.60)	**0.002**	1.31 (1.08, 1.59)	**0.007**	1.01 (0.84, 1.23)	0.905		
Age	1.00 (0.99, 1.02)	0.630			1.01 (1.00, 1.02)	0.058		
Smoking								
Never smoker	1.00		1.00		1.00			
Current smoker	1.51 (1.11, 2.05)	**0.009**	1.46 (1.05, 2.02)	**0.023**	1.14 (0.85, 1.52)	0.392		
Ex-smoker	1.28 (1.05, 1.56)	**0.013**	1.22 (0.99, 1.50)	0.062	1.31 (1.04, 1.64)	**0.021**	1.22 (0.95, 1.55)	0.114
Education								
College/university	1.00				1.00			
Primary school	1.25 (0.94, 1.66)	0.127			1.59 (0.97, 2.59)	0.065		
Secondary school	1.04 (0.86, 1.28)	0.641			1.03 (0.85, 1.26)	0.756		
Comorbidity								
Hypertension	0.93 (0.75, 1.15)	0.505			1.42 (0.89, 2.28)	0.140		
Stroke	1.03 (0.52, 2.06)	0.930			–	–		
Ischemic heart	1.88 (1.23, 2.86)	**0.003**	1.54 (0.96, 2.46)	0.071	–	–		
Diabetes	1.64 (1.09, 2.46)	**0.018**	1.46 (0.96, 2.24)	0.077	1.07 (0.47, 2.48)	0.868		
COPD	1.75 (1.07, 2.87)	**0.026**	1.49 (0.89, 2.50)	0.129	–	–		
Serious childhood infection < 5 years	1.12 (0.99, 1.27)	0.080			1.11 (0.98, 1.25)	0.106		
Respiratory symptoms								
Wheeze	1.32 (1.07, 1.63)	**0.010**	1.02 (0.72, 1.46)	0.902	1.58 (1.26, 1.97)	**< 0.001**	1.60 (1.21, 2.11)	**0.001**
Wheeze with shortness of breath	1.47 (1.15, 1.89)	**0.002**	1.22 (0.81, 1.84)	0.343	1.37 (0.92, 2.04)	0.127		
Awoken with tightness in chest	1.01 (0.76, 1.36)	0.924			1.16 (0.87, 1.53)	0.313		
Awoken with attack of breathlessness	1.26 (0.89, 1.80)	0.193			1.11 (0.72, 1.72)	0.634		
Awoken with attack of cough	1.28 (1.05, 1.55)	**0.013**	1.25 (1.00, 1.57)	**0.046**	0.94 (0.76, 1.16)	0.557		
Currently taking asthma medication	1.32 (1.00, 1.76)	0.058			1.42 (1.06, 1.91)	**0.020**	0.95 (0.60, 1.49)	0.823
Age of onset								
Early onset	–	–			1.12 (0.78, 1.62)	0.528		
Late onset	–	–			1.76 (1.32, 2.34)	**< 0.001**	1.45 (1.01, 2.07)	**0.042**

^a^Adjusted for all predictors that were significant in the univariate analyses as well as for study centre and sibling status

The statistically significant predictors from the univariate analyses were included in the multivariate logistic regression. After adjustment, the only predictors associated with reporting incorrectly for the parent-reported offspring asthma were gender (OR 1.31 for fathers versus mothers, 95% CI 1.08–1.59) and current smoker (OR 1.46, 1.05–2.02). For the offspring-reported parental asthma, only wheeze was associated with reporting incorrectly in the multivariate model (OR 1.60, 1.21–2.11). No factors were associated with reporting correctly for either the parent-reported offspring asthma or the offspring-reported parental asthma.

Further inspection of the discrepant answers given by offspring with wheeze, showed that they reported both asthma in non-asthmatic parents as well as *no* asthma in asthmatic parents slightly more often than offspring with no wheeze (Table 4). Fathers and smoking parents, on the other hand, (See Additional file 1: Table S4) showed that fathers and current smokers were more likely to report *no* asthma in asthmatic offspring than the mothers and never-smokers were. With regard to reporting asthma in non-asthmatic offspring, however, the fathers and current smokers were as correct as the mothers and never-smokers.

**Table 4:** Frequency (absolute and relative) of discrepant asthma reports according to wheezing/non-wheezing

Discrepant asthma reports	N (%)
Offspring with wheeze reporting asthma in their non-asthmatic mothers	31/655 (4.7)
Offspring without wheeze reporting asthma in their non-asthmatic mothers	94/3091 (3.0)
Offspring with wheeze reporting no asthma in their asthmatic mothers	33/655 (5.0)
Offspring without wheeze reporting no asthma in their asthmatic mothers	117/3091 (3.8)
Offspring with wheeze reporting asthma in their non-asthmatic fathers	22/510 (4.3)
Offspring without wheeze reporting asthma in their non-asthmatic fathers	52/2483 (2.1)
Offspring with wheeze reporting no asthma in their asthmatic fathers	24/510 (4.7)
Offspring without wheeze reporting no asthma in their asthmatic fathers	83/2483 (3.3)

## Discussion

In this study, agreement between self-reported asthma and asthma reported by family-members were moderate to good. The specificity was high in both offspring reports of parental asthma and parent reports of offspring asthma, suggesting a high fraction of non-asthmatics correctly identified as such by their relatives. Conversely, the sensitivity was lower for all groups, especially for the late onset asthma in the offspring i.e. a lower fraction of those with asthma after 10 years of age are correctly identified as asthmatics by their parents. The same trend was observed for the offspring; a lower fraction of asthmatic parents are correctly identified as asthmatics, while a higher fraction of the non-asthmatic parents are correctly identified. Overall, however, the vast majority of parents and offspring were in accordance with each other when reporting each other’s asthma status. In multivariate analyses, never smokers and mothers were more likely to report offspring asthma correctly. In offspring, wheeze was associated with incorrect reports of parental asthma status.

Our results showed that parents seem to have more knowledge about the asthma status of their offspring than the offspring have about their parents’ asthma condition. This may be reasonable if we assume that parents are in general more concerned with their children’s health than the children are with their parents’ health. In addition, the offspring’s awareness of the respiratory health of their parents likely depends on the severity of the parents’ asthma. Where asthma in the past was a disease with severe exacerbations, it is today a disease mostly without hospital admissions, indicating that parental asthma may be “invisible” for the offspring.

To our knowledge this is the first agreement study comparing generational reports on each other’s adult asthma status in general. Previous studies have only addressed parent-reports of current offspring asthma and not vice versa, and the children have been young [1, 23–25]. The observed high validity in parent-reported offspring asthma is in accordance with for instance a study by Cornish et al. [26] comparing parent-reported asthma to electronic patient records. That parent-reports of children’s asthma has high specificity although not so high sensitivity has also been observed in a relatively recent Canadian study [24]. High validity in parent reports of offspring atopic disease status has also been reported [27].

Cohen’s kappa and sensitivity were both lower for late onset asthma compared to early onset asthma, which may be explained by the fact that parents are more aware of their offspring’s health while they are young and still living at home. The impact on the parents and their ability to recall may be less when an offspring is diagnosed with asthma as a grownup. This is rendered particularly likely with a disease such as asthma, which is not continuously visible, but comes in attacks of various intervals – sometimes with a substantial amount of time between each attack.

In the same manner, it is likely to suspect offspring reporting their parents’ asthma more correctly if they witnessed asthma in the parents during childhood when they saw their parents on a daily basis, then if the parents developed asthma after the offspring had grown up and left home. This suspicion was confirmed in the additional analysis (See Additional file 1: Table S3), where we analysed separately offspring-reported parental asthma with onset *after* the offspring was 20 years old, and with onset *before* the offspring was 20 years old. Offspring reported asthma in their parents more correctly if the parents had their asthma during the offspring’s childhood.

We found that never-smokers and mothers were more likely to report offspring asthma correctly. Non-smokers have higher health-risk awareness than smokers [28], thus it is plausible that this group also have more knowledge regarding their offspring health. That mothers report more correctly than fathers may be explained by them generally being the primary care givers of children and consequently spending more time with them in the daily life. Today this is probably not as rigid a pattern as before, but the present agreement study is based on a parent population born between 1945 and 1973, and their offspring born between 1963 and 1997.

Wheeze in the offspring is a significant predictor for incorrect reports of parental asthma. One could speculate that offspring with wheeze over-report asthma in their parents because of a hyper alertness concerning asthma and its symptoms. This would be in accordance with what has been observed for instance in atopic diseases, where fathers were more likely to recall their own atopic disease history if their children currently had severe atopic diseases [29]. In addition, Danell et al. [30] showed that children reported more asthma-related issues than their parents regarding their own symptoms. However, in our study, the discrepant answers given by offspring with wheeze were associated with both under-reporting and over-reporting (See Additional file 1: Table S4), and further studies are needed to appropriately investigate the mechanisms behind these discrepancies. Although seemingly random, the observed misclassification may lead to a bias towards the null and yield weaker associations than what actually exist in real life.

Regarding the parents, we observed an increased risk for differential misclassification of offspring asthma among men and smokers, with a tendency for under-estimating asthma in their offspring. As suspected, we also observed that this type of misclassification was more widespread regarding late onset asthma in the offspring than early onset asthma. The same pattern was also observed for offspring reporting parental asthma with onset after the offspring had grown up and left home.

Apart from male gender and current smoking in parents, and wheeze in offspring, we did not observe any other significant predictors for discrepant asthma reports across generations. This leads us to believe that discrepant reports concerning offspring health status are mostly due to minor and random misclassification with little consequences for the validity of asthma reports.

Nevertheless, the observed predictors for discrepant answers should be taken into consideration in any future studies relying on asthma reports on behalf of family members.

The main strengths of this study are population size and study design; to our knowledge RHINESSA is the largest population-based generational study so far, and this is the first study to compare self-report questionnaires across generations. In addition to the agreement analyses, we also identified predictors for disagreement, which was possible through the large dataset collected in the RHINE, ECRHS and RHINESSA.

However, some limitations to our study should also be acknowledged. First, the response rate in the offspring population was low: Only a third of the offspring of ECRHS/RHINE participants agreed to participate in the RHINESSA generation study. However, the offspring population was not severely skewed in any direction – distribution of demographic characteristics such as sex, smoking habits and educational level did not seem to differ substantially from that of a general population in the same age range. We also examined distribution of sex, smoking habits, educational level and asthma status of parents with participating offspring and parents with non-participating offspring to further examine any potential response bias. Our data showed that the groups were very similar. There was a miniscule overrepresentation in RHINESSA of offspring who had parents with asthma (13.6% versus 11.5% in non-participating offspring), parents who were non-smokers (84.3% versus 80.6%), and female parents (mothers, 55.3% versus 50.6%). In addition, slightly less parents with higher education had offspring who participated in RHINESSA (46.1% versus 47.9% of the parents with non-participating offspring). These very small differences are reassuring, and do not provide any evidence of differential misclassification due to response bias. In addition, we were not aiming to assess disease prevalence in the present study, but to assess associations between two variables (parent reports and offspring reports). Although a low response rate may have impact on prevalence rates, internal exposure-outcome associations are less affected [20, 31]. However, we cannot be entirely certain that the observed differences in agreement between centres (Additional file 1: Table S2a and b), are valid for the general populations in these centres or if they apply only to the select group of participants.

Secondly, self-report questionnaires are susceptible to misclassification in the form of recall bias, especially if the outcomes date far back in time. Through comparing offspring-reports on parental asthma and parent-reports on offspring asthma, we set as a prerequisite that the reports they have given regarding themselves are correct. A comparison with primary care records for the study participants would have helped us assess presence or absence of recall bias in our study. Other possibilities would be to use prescription registry data. Data on dispensed antiasthmatics from the Norwegian Prescription Database (NorPD) has previously been validated in the Norwegian mother and child cohort study (MoBa), and they found that the use of prescription data for 7-year old children had high validity [32]. Unfortunately, such data was not available in our study. However, while clinical assessment is often considered the best method for validating self-reported asthma, recent studies presented self-report questionnaires to be a reliable tool in an epidemiological setting [8, 26].

Thirdly, we used “doctor-diagnosed asthma” when subjects reported about themselves, but “ever asthma” when subjects reported about each other. A recent Danish study showed how asthma prevalence in children is highly dependent on the method of measuring asthma, being lowest when measured with hospitalization data and highest when measured with prescription data, and with self-reports by parents in between the two other methods [4].

Since the asthma report on behalf of the other generation in our study was ever asthma and not doctor-diagnosed, one could suspect an over-report of asthma on behalf of the other generation. However, the results of our study did not show any such tendency. This is in line with previous research by de Marco et al., who found that the question “Do you have or have you ever had asthma?” gave prevalence estimates comparable to clinical diagnosis [33].

Furthermore, in RHINESSA only one of the parents is included, while ideally both parents should have been included. However, mothers and fathers were approximately equally distributed in RHINESSA and consequently we have no reason to believe that there is a gender bias present.

## Conclusion

In conclusion, this agreement study shows a moderate to good agreement between the self-reported asthma and asthma reported by family-members, although we observed some risk of under-report. In the absence of direct reports, offspring asthma status reported by parents and parental asthma status reported by offspring may be used as a proxy, both in epidemiological studies and in a clinical setting before undergoing further clinical examination.

## Additional file

Additional file 1: **Table S1.** Response rates of the offspring population, RHINESSA. **Table S2a and 2b.** Parameter estimates and 95% confidence intervals for Cohen’s kappa, sensitivity, specificity, positive predictive value (PPV), and negative predictive value (NPV) for offspring-reported parental asthma and parent- reported offspring asthma, stratified by study centers. **Table S3.** Parameter estimates and 95% confidence intervals for sensitivity, specificity, positive predictive value (PPV), and negative predictive value (NPV) for offspring-reported parental asthma and parent-reported offspring asthma, according to timing of parental asthma onset. **Table S4.** Frequency (absolute and relative) of discrepant asthma reports according to fathers/mothers, and current smoking parents/never-smoking parents. (DOCX 36 kb)

## Abbreviations

CI: Confidence interval
COPD: Chronic obstructive lung disease
ECRHS: European Community Respiratory Health Survey
MoBa: Norwegian mother and child cohort study
NorPD: The Norwegian Prescription Database
NPV: Negative predictive value
OR: Odds ratio
PPV: Positive predictive value
RHINE: Respiratory Health in Northern Europe
RHINESSA: Respiratory Health in Northern Europe, Spain and Australia
