# Supplementary material for: Agreement in reporting of asthma by parents or offspring – the RHINESSA generation study
Source: BMC Pulm Med. 2018 Jul 27;18:122. doi: 10.1186/s12890-018-0687-4 (PMC6062946; doi:10.1186/s12890-018-0687-4)
Supplement: Supplementary file 1 — Table S1. Response rates of the offspring population, RHINESSA. Table S2a and 2b. Parameter estimates and 95% confidence intervals for Cohen’s kappa, sensitivity, specificity, positive predictive value (PPV), and negative predictive value (NPV) for offspring-reported parental asthma and parent- reported offspring asthma, stratified by study centers. Table S3. Parameter estimates and 95% confidence intervals for sensitivity, specificity, positive predictive value (PPV), and negative predictive value (NPV) for offspring-reported parental asthma and parent-reported offspring asthma, according to timing of parental asthma onset. Table S4. Frequency (absolute and relative) of discrepant asthma reports according to fathers/mothers, and current smoking parents/never-smoking parents. (DOCX 36 kb) [file 12890_2018_687_MOESM1_ESM.docx]

Additional files.

Table S1. Response rates of the offspring population, RHINESSA.

| **Country (center)** | **Response rate (%)** |
| --- | --- |
| Denmark (Aarhus) | 30.5 |
| Iceland (Reykjavik) | 25.0 |
| Norway (Bergen) | 40.1 |
| Sweden (Gothenburg, Umea, Uppsala) | 43.7 |
| Estonia (Tartu) | 18.6 |
| Spain (Albacete, Huelva) | 24.6 |
| Australia (Melbourne) | 73.7 |

Table S2a and b. Parameter estimates and 95% confidence intervals for Cohen’s Kappa, sensitivity, specificity, PPV, and NPV for offspring-reported parental asthma and parent- reported offspring asthma, stratified by study centers.

| **a) Offspring asthma** | **Agreement^1^ N (%)** | **Disagreement^2^ N (%)** | **Cohen’s kappa** | **Sensitivity** | **95% CI** | **Specificity** | **95% CI** | **PPV** | **95% CI** | **NPV** | **95% CI** |
| --- | --- | --- | --- | --- | --- | --- | --- | --- | --- | --- | --- |
| *Early onset asthma* | *4845 (96)* | *204 (4)* | *0.71* | *0.66* | *0.61, 0.71* | *0.99* | *0.98, 0.99* | *0.81* | *0.76, 0.85* | *0.97* | *0.97, 0.98* |
| Aarhus | 568 (97) | 19 (3) | 0.69 | 0.68 | 0.50, 0.83 | 0.99 | 0.97, 0.99 | 0.74 | 0.55, 0.88 | 0.98 | 0.97, 0.99 |
| Reykjavik | 625 (93) | 47 (7) | 0.68 | 0.66 | 0.55, 0.75 | 0.97 | 0.96, 0.98 | 0.79 | 0.68, 0.87 | 0.95 | 0.93, 0.96 |
| Bergen | 974 (96) | 38 (4) | 0.65 | 0.57 | 0.44, 0.68 | 0.99 | 0.98, 1.00 | 0.83 | 0.69, 0.92 | 0.97 | 0.96, 0.98 |
| Gothenburg | 653 (97) | 20 (3) | 0.74 | 0.76 | 0.60, 0.88 | 0.98 | 0.97, 0.99 | 0.76 | 0.60, 0.88 | 0.98 | 0.97, 0.99 |
| Umea | 882 (96) | 39 (4) | 0.74 | 0.66 | 0.56, 0.76 | 0.99 | 0.98, 1.00 | 0.90 | 0.81, 0.96 | 0.96 | 0.95, 0.97 |
| Uppsala | 882 (96) | 35 (4) | 0.74 | 0.70 | 0.58, 0.80 | 0.99 | 0.98, 0.99 | 0.83 | 0.72, 0.91 | 0.97 | 0.96, 0.98 |
| Tartu | 284 (98) | 6 (2) | 0.56 | 0.67 | 0.22, 0.96 | 0.99 | 0.96, 1.00 | 0.50 | 0.16, 0.84 | 0.99 | 0.97, 1.00 |
| Albacete/Huelva | 108 (96) | 4 (4) | 0.83 | 0.85 | 0.55, 0.98 | 0.98 | 0.93, 1.00 | 0.85 | 0.55, 0.98 | 0.98 | 0.93, 1.00 |
| Melbourne | 145 (93) | 11 (7) | 0.83 | 0.79 | 0.65, 0.90 | 0.99 | 0.95, 1.00 | 0.97 | 0.87, 1.00 | 0.92 | 0.85, 0.96 |
| *Late onset asthma* | *4750 (94)* | *304 (6)* | *0.48* | *0.37* | *0.33, 0.42* | *0.99* | *0.99, 0.99* | *0.78* | *0.72, 0.84* | *0.95* | *0.94, 0.95* |
| Aarhus | 313 (97) | 11 (3) | 0.55 | 0.60 | 0.39, 0.79 | 1.00 | 0.98, 1.00 | 0.94 | 0.70, 1.00 | 0.97 | 0.94, 0.98 |
| Reykjavik | 597 (93) | 46 (7) | 0.45 | 0.34 | 0.23, 0.48 | 0.99 | 0.98, 1.00 | 0.78 | 0.58, 0.91 | 0.94 | 0.91, 0.95 |
| Bergen | 962 (93) | 74 (7) | 0.42 | 0.33 | 0.24, 0.44 | 0.99 | 0.98, 0.99 | 0.72 | 0.56, 0.85 | 0.94 | 0.92, 0.95 |
| Gothenburg | 647 (94) | 41 (6) | 0.47 | 0.36 | 0.23, 0.50 | 0.99 | 0.98, 1.00 | 0.80 | 0.59, 0.93 | 0.95 | 0.93, 0.96 |
| Umea | 857 (90) | 91 (10) | 0.43 | 0.34 | 0.25, 0.43 | 0.99 | 0.98, 0.99 | 0.80 | 0.67, 0.90 | 0.91 | 0.89, 0.93 |
| Uppsala | 864 (93) | 65 (7) | 0.47 | 0.35 | 0.25, 0.46 | 0.99 | 0.98, 1.00 | 0.84 | 0.69, 0.94 | 0.93 | 0.92, 0.95 |
| Tartu | 198 (99) | 2 (1) | 0.61 | 0.60 | 0.15, 0.95 | 1.00 | 0.98, 1.00 | 1.00 | 0.29, 1.00 | 0.99 | 0.96, 1.00 |
| Albacete/Huelva | 101 (86) | 16 (14) | 0.39 | 0.39 | 0.17, 0.64 | 0.95 | 0.89, 0.98 | 0.58 | 0.28, 0.85 | 0.90 | 0.82, 0.95 |
| Melbourne | 116 (93) | 9 (7) | 0.63 | 0.53 | 0.28, 0.77 | 0.99 | 0.95, 1.00 | 0.90 | 0.28, 0.85 | 0.93 | 0.87, 0.97 |

| **b) Parental asthma** | **Agreement^1^ N (%)** | **Disagreement^2^ N (%)** | **Cohen’s kappa** | **Sensitivity** | **95% CI** | **Specificity** | **95% CI** | **PPV** | **95% CI** | **NPV** | **95% CI** |
| --- | --- | --- | --- | --- | --- | --- | --- | --- | --- | --- | --- |
| *Maternal* | *3347 (93)* | *255 (7)* | *0.68* | *0.70* | *0.65, 0.74* | *0.96* | *0.96, 0.97* | *0.74* | *0.70, 0.78* | *0.96* | *0.95, 0-96* |
| Aarhus | 357 (95) | 18 (5) | 0.78 | 0.78 | 0.63, 0.88 | 0.98 | 0.96, 0.99 | 0.84 | 0.71, 0.94 | 0.97 | 0.94, 0.98 |
| Reykjavik | 412 (88) | 56 (12) | 0.54 | 0.55 | 0.44, 0.66 | 0.95 | 0.92, 0.97 | 0.69 | 0.56, 0.80 | 0.91 | 0.88, 0.94 |
| Bergen | 565 (92) | 50 (8) | 0.66 | 0.64 | 0.54, 0.74 | 0.97 | 0.95, 0.98 | 0.80 | 0.69, 0.88 | 0.94 | 0.91, 0.96 |
| Gothenburg | 487 (96) | 22 (4) | 0.76 | 0.78 | 0.65, 0.89 | 0.98 | 0.96, 0.99 | 0.78 | 0.65, 0.89 | 0.98 | 0.96, 0.99 |
| Umea | 690 (94) | 43 (6) | 0.74 | 0.79 | 0.70, 0.87 | 0.96 | 0.94, 0.98 | 0.75 | 0.66, 0.84 | 0.97 | 0.95, 0.98 |
| Uppsala | 673 (92) | 55 (8) | 0.66 | 0.69 | 0.59, 0.78 | 0.96 | 0.94, 0.97 | 0.71 | 0.61, 0.80 | 0.95 | 0.94, 0.97 |
| Tartu | 189 (93) | 15 (7) | 0.48 | 0.62 | 0.32, 0.86 | 0.95 | 0.91, 0.98 | 0.44 | 0.22, 0.69 | 0.97 | 0.94, 0.99 |
| Albacete/Huelva | 89 (82) | 20 (18) | 0.51 | 0.65 | 0.44, 0.83 | 0.87 | 0.78, 0.93 | 0.61 | 0.41, 0.79 | 0.89 | 0.80, 0.95 |
| Melbourne | 34 (97) | 1 (3) | 0.65 | 1.00 | 0.89, 1.00 | 0.50 | 0.01, 0.99 | 0.97 | 0.85, 1.00 | 1.00 | 0.03, 1.00 |
| *Paternal* | *2803 (93)* | *193 (7)* | *0.67* | *0.64* | *0.58, 0.69* | *0.97* | *0.96, 0.98* | *0.70* | *0.64, 0.75* | *0.96* | *0.95, 0.97* |
| Aarhus | 308 (96) | 13 (4) | 0.74 | 0.72 | 0.53, 0.87 | 0.98 | 0.96, 0.99 | 0.81 | 0.61, 0.93 | 0.97 | 0.95, 0.99 |
| Reykjavik | 373 (89) | 44 (11) | 0.38 | 0.35 | 0.22, 0.51 | 0.97 | 0.94, 0.98 | 0.57 | 0.37, 0.75 | 0.92 | 0.89, 0.95 |
| Bergen | 532 (93) | 39 (7) | 0.69 | 0.64 | 0.53, 0.75 | 0.98 | 0.96, 0.99 | 0.84 | 0.72, 0.92 | 0.94 | 0.92, 0.96 |
| Gothenburg | 407 (97) | 14 (3) | 0.76 | 0.77 | 0.59, 0.90 | 0.98 | 0.96, 0.99 | 0.77 | 0.59, 0.90 | 0.98 | 0.96, 0.99 |
| Umea | 511 (93) | 36 (7) | 0.60 | 0.60 | 0.45, 0.73 | 0.97 | 0.95, 0.98 | 0.67 | 0.52, 0.81 | 0.96 | 0.94, 0.97 |
| Uppsala | 517 (96) | 24 (4) | 0.77 | 0.87 | 0.75, 0.95 | 0.97 | 0.95, 0.98 | 0.73 | 0.60, 0.83 | 0.99 | 0.97, 0.99 |
| Tartu | 88 (96) | 4 (4) | 0.48 | 0.40 | 0.05, 0.85 | 0.99 | 0.94, 1.00 | 0.67 | 0.09, 0.99 | 0.97 | 0.91, 0.99 |
| Albacete/Huelva | 86 (85) | 15 (15) | 0.58 | 0.54 | 0.34, 0.73 | 0.97 | 0.91, 1.00 | 0.88 | 0.64, 0.99 | 0.85 | 0.75, 0.92 |
| Melbourne | 29 (88) | 4 (12) | 0.68 | 1.00 | 0.85, 1.00 | 0.60 | 0.26, 0.88 | 0.85 | 0.66, 0.96 | 1.00 | 0.54, 1.00 |

^1^ Agreement: When both parents and offspring answered the same (yes/yes or no/no).

^2^ Disagreement: When parents and offspring answered differently (yes/no or no/yes).

Table S3. Parameter estimates and 95% confidence intervals for sensitivity, specificity, PPV, and NPV for offspring-reported parental asthma and parent-reported offspring asthma, according to timing of parental asthma onset.

| **Parental asthma** | **Agreement^1^ N (%)** | **Disagreement^2^ N (%)** | **Sensitivity** | **95% CI** | **Specificity** | **95% CI** | **PPV** | **95% CI** | **NPV** | **95% CI** |
| --- | --- | --- | --- | --- | --- | --- | --- | --- | --- | --- |
| Asthma onset in mothers < offspring age 20 | 3982 (91) | 371 (9) | 0.75 | 0.70, 0.79 | 0.93 | 0.92, 0.94 | 0.52 | 0.48, 0.56 | 0.97 | 0.97, 0.98 |
| Asthma onset in mothers > offspring age 20 | 3814 (88) | 539 (12) | 0.61 | 0.52, 0.70 | 0.89 | 0.88, 0.89 | 0.14 | 0.12, 0.18 | 0.99 | 0.98, 0.99 |
| Asthma onset in fathers < offspring age 20 | 3351 (94) | 233 (6) | 0.72 | 0.66, 0.77 | 0.95 | 0.95, 0.96 | 0.58 | 0.53, 0.63 | 0.97 | 0.97, 0.98 |
| Asthma onset in fathers > offspring age 20 | 3224 (90) | 360 (10) | 0.48 | 0.33, 0.63 | 0.91 | 0.90, 0.92 | 0.06 | 0.04, 0.09 | 0.99 | 0.99, 1.00 |

^1^ Agreement: When both parents and offspring answered the same (yes/yes or no/no).

^2^ Disagreement: When parents and offspring answered differently (yes/no or no/yes).

Table S4. Frequency (absolute and relative) of discrepant asthma reports according to fathers/mothers, and current smoking parents/never-smoking parents.

| **Discrepant asthma reports** | **N (%)** |
| --- | --- |
| Fathers reporting early onset asthma in early non-asthmatic offspring | 28/2293 (1.2) |
| Mothers reporting early onset asthma in early non-asthmatic offspring | 39/3047 (1.3) |
| Fathers reporting no asthma in offspring with early onset asthma | 80/2293 (3.5) |
| Mothers reporting no asthma in offspring with early onset asthma | 72/3047 (2.4) |
| Fathers reporting late onset asthma in late non-asthmatic offspring | 21/2285 (0.9) |
| Mothers reporting late onset asthma in late non-asthmatic offspring | 28/3092 (0.9) |
| Fathers reporting no asthma in offspring with late onset asthma | 156/2285 (6.8) |
| Mothers reporting no asthma in offspring with late onset asthma | 171/3092 (5.5) |
| Current smoking parents reporting early onset asthma in early non-asthmatic offspring | 5/466 (1.1) |
| Never-smoking parents reporting early onset asthma in early non-asthmatic offspring | 25/2413 (1.0) |
| Current smoking parents reporting no asthma in offspring with early onset asthma | 13/466 (2.9) |
| Never-smoking parents reporting no asthma in offspring with early onset asthma | 59/2413 (2.4) |
| Current smoking parents reporting late onset asthma in late non-asthmatic offspring | 7/483 (1.4) |
| Never-smoking parents reporting late onset asthma in late non-asthmatic offspring | 20/2433 (0.8) |
| Current smoking parents reporting no asthma in offspring with late onset asthma | 39/483 (8.1) |
| Never-smoking parents reporting no asthma in offspring with late onset asthma | 129/2433 (5.3) |
